# Electroencephalogram and phenotype patterns in neuronopathic Gaucher disease patients – ten years of experience in a single center

**DOI:** 10.1186/s42494-024-00168-1

**Published:** 2024-09-01

**Authors:** Xiying Yang, Yan Meng, Jian Chen, Qian Lu, Linyan Hu, Xiuyu Shi, Jing Wang, Guang Yang, Liping Zou

**Affiliations:** Department of Pediatrics, the First Center of General Hospital of the People’s Liberation Army, Fuxing Road, Haidian District, Beijing, 100853 No 28 China

**Keywords:** Gaucher disease, Neuronopathic, Electroencephalogram, Epilepsy, Anti-seizure medication, Enzyme replacement therapy

## Abstract

**Background:**

This study aimed to investigate the unique electroencephalography (EEG) patterns in neuronopathic Gaucher disease (GD) patients and explore the correlations between EEG findings and neurological phenotypes so as to optimize clinical outcomes.

**Methods:**

A retrospective analysis was conducted on 74 EEG recordings from 50 GD patients between January 2012 and July 2022.

**Results:**

Twenty-three patients exhibited abnormal EEG recordings, including 11 of the GD1 type (the transitional type) and 12 with neuronopathic GD. Of the 12 neuronopathic GD patients, 9 patients with epilepsy were analysed specifically in terms of the clinical course. The primary waveform observed in the neuronopathic EEG recordings was the spike-and-wave complex (SWC) during both awake and sleep states. This was significantly different from sharp waves observed only during sleep in the patients of the transitional type (*P* = 0.0230). The abnormal discharges in the neuronopathic patients were most commonly located in the bilateral Rolandic areas, while the transitional type commonly involved the bilateral frontal regions. Three patients with an epileptic EEG pattern reported their initial seizures years later. Seizures in the neuronopathic patients were effectively controlled with anti-seizure medications (ASMs), despite the ongoing presence of abnormal EEG patterns. The EEG patterns during ocular symptoms were characterized by sporadic or continuous unilateral SWC during sleep.

**Conclusions:**

Patients with neuronopathic GD exhibit distinct EEG patterns that can help differentiate them from GD1 patients. Early treatment with ASMs can effectively control seizures. EEG plays a crucial role in monitoring seizures and can facilitate prompt intervention for GD patients.

## Background

Type 3 Gaucher disease (GD3), component of neuronopathic type of Gaucher disease (GD) together with GD2, is a common clinical type in certain countries in Northeast Asia and Western Europe. The prevalence of neuronopathic GD is high in countries such as Sweden, Poland, China and Japan, and is estimated to reach 30–50% of all GD patients [1, 2], which is consistent to the statistics in our center. The worldwide prevalence of GD is about 1.33–1.75 per 100,000 [3], thus the minimum prevalence of neuronopathic GD would be 0.44–0.58 per 100,000. The neuronopathic type of GD is difficult to manage and has a poor prognosis compared to GD1 [4, 5], significantly impairing the overall survival and quality of life of GD patients. Clinical efforts are focused on preventing or delaying the onset of irreversible neurological symptoms such as epilepsy. Early diagnosis, regular monitoring, and anti-seizure medications (ASMs) are crucial for the management of seizures.

Epilepsy is one of the most common neurological manifestations in GD patients and can be managed with medication [6]. The severity of seizures varies. In some cases, GD may progress to more severe forms like progressive myoclonic epilepsy (PME), which may become intractable, leading to cognitive deficits and cerebral and/or cerebellar atrophy [4, 7]. Therefore, achieving a seizure-free status is of great clinical significance for improving the prognosis and the quality of life of neuronopathic GD patients. Electroencephalogram (EEG) monitoring plays a crucial role in diagnosing, classifying, and evaluating treatment efficacy in GD patients with epilepsy [6, 8, 9]. EEG is a potential tool for early recognition of GD1 patients progressing to GD3, allowing for timely interventions such as ASMs upon seizure onset.

However, the value of EEG for GD patients is currently underestimated. There have been limited studies on EEG use in GD patients over the past four decades [9, 10]. The correlations of EEG features with clinical phenotypes, interventions, and outcomes remain unclear. In this study, we set out to address the following questions by analyzing 74 EEG monitoring records from 50 GD patients over the past 10 years at our center:

1. Are there distinct EEG patterns in neuronopathic GD patients that can predict the progression from GD1 to GD3?
2. Is there a correlation between EEG patterns and neurological clinical phenotypes?
3. How can we optimize clinical benefits by incorporating EEG monitoring with different interventions?

Clarifying these questions can enhance our understanding of the EEG patterns in GD as well as their associations with clinical phenotypes, and ultimately improve patient outcomes through effective targeted interventions with utilization of EEG monitoring.

## Methods

### Source of study data

The Pediatric Department of General Hospital of the People's Liberation Army is a specialized medical center for the diagnosis and treatment of rare diseases, including GD, and serves as an epilepsy research center as well. In this retrospective study, 50 GD patients with definitive genetic and enzymological diagnosis and having received regular follow-ups were included. This study involved the expertise of child neurologists, epilepsy professors, and GD experts, ensuring a comprehensive and multidisciplinary approach to the research.

All patients’ parents or guardians had given informed consent. The study was approved by the hospital Ethics Committee.

### Measurement

In our study, epilepsy in GD patients was defined as the occurrence of more than one afebrile convulsion after excluding infectious, inflammatory, or other potential causes, coupled with observation of EEG abnormalities. The classification and evaluation of the effectiveness of ASMs against seizures were based on the guidelines provided by the International League Against Epilepsy (ILAE) in 2017. The overall outcome in terms of seizure control was also categorized based on these guidelines.

Both video EEG (VEEG) and ambulatory EEG (AEEG) monitoring were conducted at least once in our center. For VEEG, 19-lead recording electrodes were placed according to the international 10–20 system. Each monitoring lasted at least 1 h, comprising a complete recording of the awake-sleep cycle. The frequency of EEG recordings varied among individuals due to various practical factors, such as economic, pandemic and clinical considerations, with intervals from weeks to years. EEG monitoring and reporting was carried out by the same team of experienced EEG technicians and child electrophysiologists, who were blinded to the clinical types and restricted to only knowing the GD diagnosis.

### Statistical analysis

**Fig. 1:**
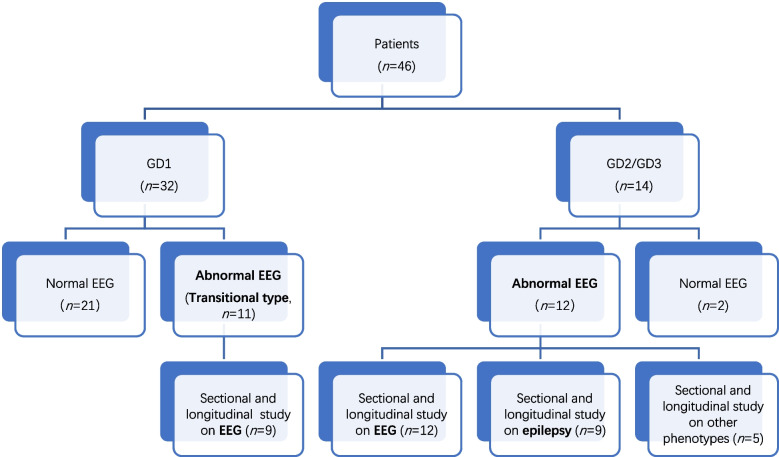
Workflow of the study. Seventy-four EEG recordings from 46 GD patients were analyzed. Patients with EEG abnormalities were followed up for EEG or epilepsy attacks

In this cross-sectional EEG study, the most recent EEG recording of each patient was used for comparison to characterize EEG patterns across different clinical types. Additionally, within the scope of the EEG longitudinal study, we specifically focused on patients who had undergone two or more EEG recordings to observe and document the evolution of their EEG patterns. The workflow of this study is shown in Fig. 1. The quantitative data are expressed as median ± standard deviation.

The EEG background, sleep-awake cycle, abnormal wave locations, waveform, wave amplitude and clinical attacks were analyzed with the Graphpad Prism 8.0 software. Normal distribution of the data was verified before the test. Differences between two groups were analyzed with *t*-test or Fisher-exact test. *P* < 0.05 was considered statistically significant.

## Results

Forty-six paediatric GD patients were included in this retrospective study between January 2012 and July 2022, comprising 26 males and 20 females, with a sexual ratio of 1.3∶1. Three cases with incomplete data and one case with EEG recording from another hospital were excluded from the analysis. Of the remaining patients, 14 were diagnosed with the neuronopathic type (one GD2 and the rest GD3), of which 12 displayed EEG abnormalities (85.7%) (Table 1). Among the 32 non-neuronopathic cases (GD1), 11 (34.4%) showed EEG abnormalities (Fig. 1). The GD1 patients with abnormal EEG recordings were defined as the “transitional type” (Fig. 1).

**Table 1:** The clinical summary of GD patients whose EEG monitoring had neuronopathic features

**Pt’s No**	**Age at time of EEG**	**GD type**	**Seizure types**	**Eye findings**		**Other neurological symptoms**	**Major EEG abnormities**		
				**HSNP**	**STR**		**Awake**	**Discharges**	
							**Sleep**		
1	13y 7mo	3	Myoclonic Tonic-clonic	(-)	(-)	Ataxia	+ +	Polyspike and wave, spike rhythm	
2	8y 4mo	3	Tonic-clonic Tonic	(-)	+	(-)	+ +	(Poly)spike and wave, ESES	
3	16y 11mo	3	Myoclonic	(-)	(-)	Parkinsonian tremor	+ +	Polyspike and wave	
4	19y 9mo	3	Tonic-clonic	(-)	(-)	(-)	+ +	(Poly)spike and wave	
5	8mo	2	Tonic	(-)	(-)	Developmental delay	(-) +	Spike and wave	
6	5y 2mo	3	Tonic-clonic Clonic	+	+	Ataxia, Developmental delay, Parkinsonian tremor	+ +	(Poly)spike and wave	
7	20y 2mo	3	Tonic	(-)	(-)	(-)	+ +	(Poly)spike and wave	
8	7y 10mo	3	Tonic-clonic	(-)	(-)	Ataxia	+ +	Spike and wave	
9	8y 11mo	3	Tonic	(-)	(-)	Developmental delay	+ +	(Spike)sharp and wave	
10	9y 4mo	3	(-)	(-)	+	Developmental delay, Parkinsonian tremor	+ +	Spike and wave	
11	6y	3	(-)^R^	+	(-)	Ataxia	+ +	(Poly)spike and wave	
12	4y	3	(-)	+	+	(-)	(-) +	Spike and wave	
13	11y 10mo	1	(-)^R^	(-)	(-)	(-)	+ +	Polyspike and wave, spike rhythm	
14	11y 11mo	1	(-)^R^	(-)	(-)	(-)	+ NREM I	Spike and wave	
15	8y	1	(-)	(-)	(-)	(-)	+ +	Sharp and wave	

*HSNP* Horizontal supranuclear palsy, ophthalmoplegia, *STR *Strabismus, *NREM I *Non-rapid eye movement stage I, ^*R*^ initial seizure episodes were reported when composing this article, *(-)* Abscent

In our study, a total of 23 patients (50.0%) exhibited abnormal EEG monitoring results. Among them, 12 patients were identified as the neuronopathic phenotype, with nine of them presenting primarily with epilepsy, warranting further investigation. The remaining 11 patients were classified as the transitional type (Fig. 1).

Notably, two patients from the transitional type and one GD3 patient experienced their initial seizure episodes as predicted from their EEG recording patterns.

1. Different waveforms in neuronopathic GD patients and transitional patients

The primary waveform observed in neuronopathic EEG recordings was the spike-and-wave complex (SWC) that appeared at both awake and sleep states.

**Fig. 2:**
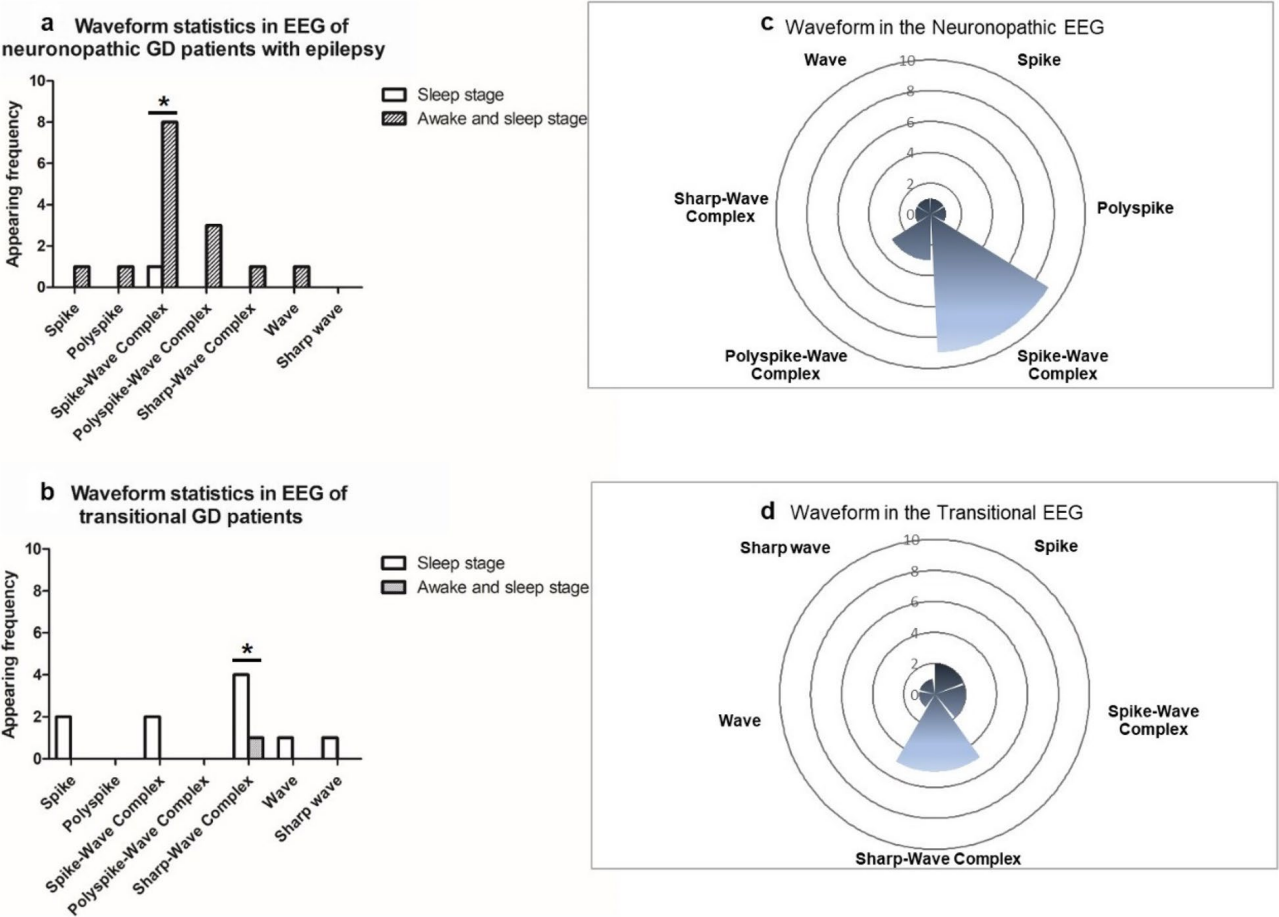
Different EEG waveform patterns of the neuronopathic and transitional types of GD patients. **a** A column diagram of EEG waveform patterns in neuronopathic patients with epilepsy during sleep and awake states; **b** A column diagram of EEG waveform patterns in transitional patients during sleep and awake states; the frequency of the spike and wave complex (SWC) and sharp and wave complex from neuronopathic and transitional EEG was significantly different (*P* = 0.023*). **c** The rose diagram of waveform pattern in the neuronopathic EEG; **d **The rose diagram of waveform patterns in the transitional EEG

In contrast, the transitional patients primarily showed sharp waves, mostly occurring during sleep (*P* = 0.0230, OR = 0.0312, 95% CI 0.0015–0.6412). The waveform type of neuronopathic EEG was typical SWC accompanied by polyspike-wave complex, with no sharp waves. The waveform type of the transitional patient EEG was characterized by the sharp-and-wave complex, with some spikes and SWCs but no polyspikes or polyspike-wave complex (Fig. 2).

(2) The “Bilateral Rolandic areas” was the common location of abnormal discharges in the neuronopathic EEG

**Fig. 3:**
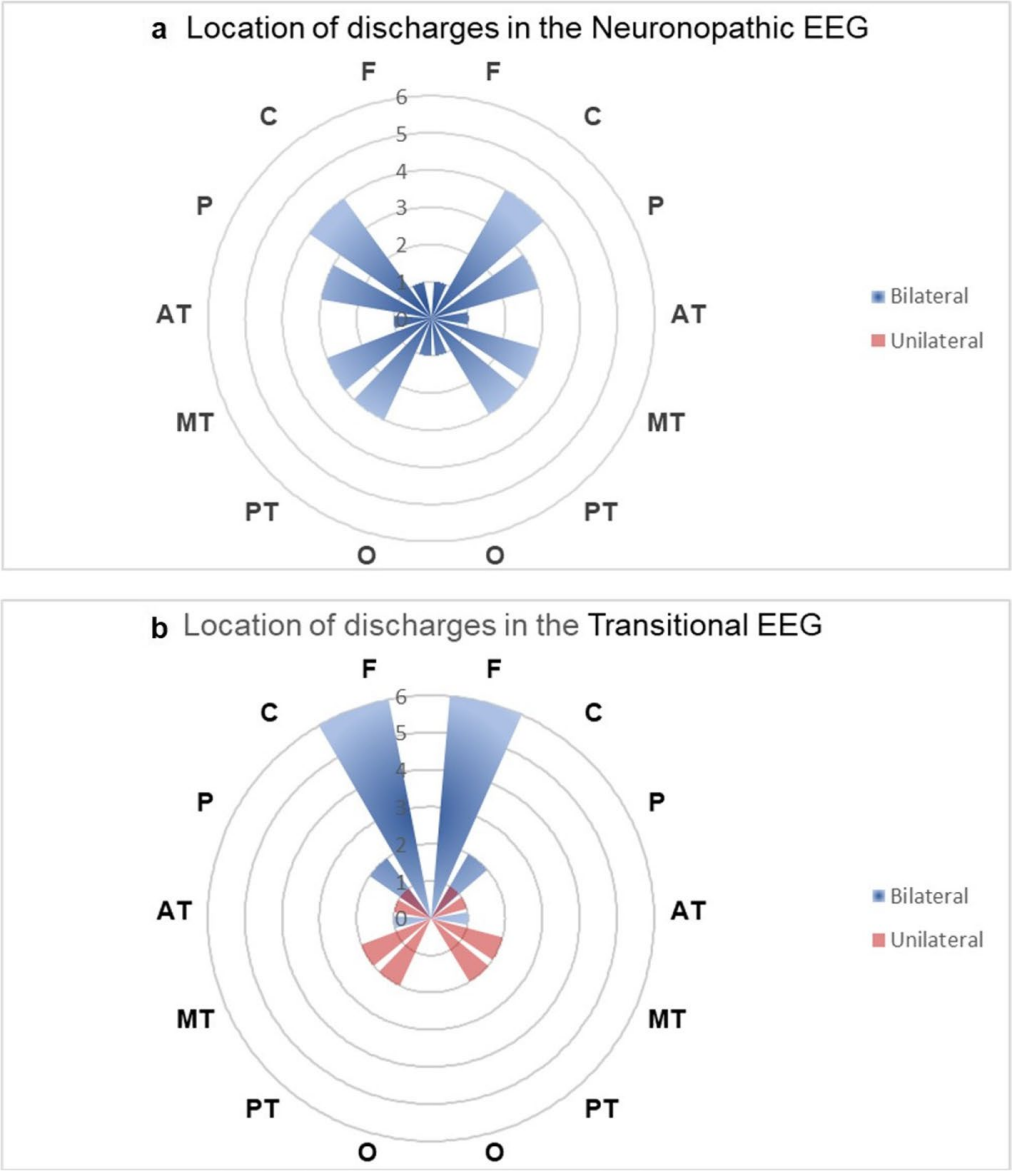
The distribution of abnormal discharges in neuronopathic and transitional EEG. **a** The location statistics of bilateral or unilateral abnormal discharges in EEG of neuronopathic patients with epilepsy; **b** The location statistics of bilateral or unilateral abnormal discharges in EEG of transitional patients. (Abbreviations: *F* Frontal, *C* Central, *P* Parietal, *AT* Anterior temporal, *MT* Medial temporal, *PT* Posterior temporal, *O* Occipital)

Statistical analysis revealed that the abnormal discharges in the neuronopathic EEG were most frequently observed in the bilateral Rolandic areas (including the central, parietal, medial temporal, and posterior temporal lobes), with occasional involvement of the bilateral occipital or frontal lobes. In contrast, the transitional GD patients primarily exhibited abnormal discharges in the bilateral frontal lobe, with less frequent involvement of the unilateral or bilateral Rolandic areas and no involvement of the occipital region (Fig. 3).

(3) Other EEG findings

The interictal EEG of the neuronopathic GD patients was characterized by generalized (3 of 12) or bilateral multi-focal (9 of 12) sporadic or continuous SWC, occasionally accompanied by the polyspike-wave complex. The amplitude of these waveforms ranged from low to median. Among the neuronopathic GD patients, 10 cases showed discharges in both sleep and awake states, while two cases only exhibited discharges during sleep. In three cases, a spike or SWC rhythm was observed, and one case presented with electrical status epilepticus during sleep (ESES). Ten cases (83.3%) showed normal background activity and sleep structure. One case showed abnormal waveforms during sleep and disappearance of occipital rhythmic activity. The other case displayed drug-induced fast activity in both resting-awake and sleep stages. Epileptic episodes were detected in three patients.

The interictal EEG of the transitional-type patients were characterized by generalized (2 of 9) or bilateral multi-focal (4 of 9) sporadic or episodic sharp waves with a median or low amplitude (ranging from low to median low). Eight cases had abnormal discharges during sleep and one case had abnormal discharges in both awake and sleep states, which was statistically different from the neuronopathic patients (*P* = 0.0019). The background activity and sleep cycle were all normal. No epileptic episode was detected.

(4) Seizures of neuronopathic patients were controlled by ASMs with various EEG evolutions in the longitudinal study

**Fig. 4:**
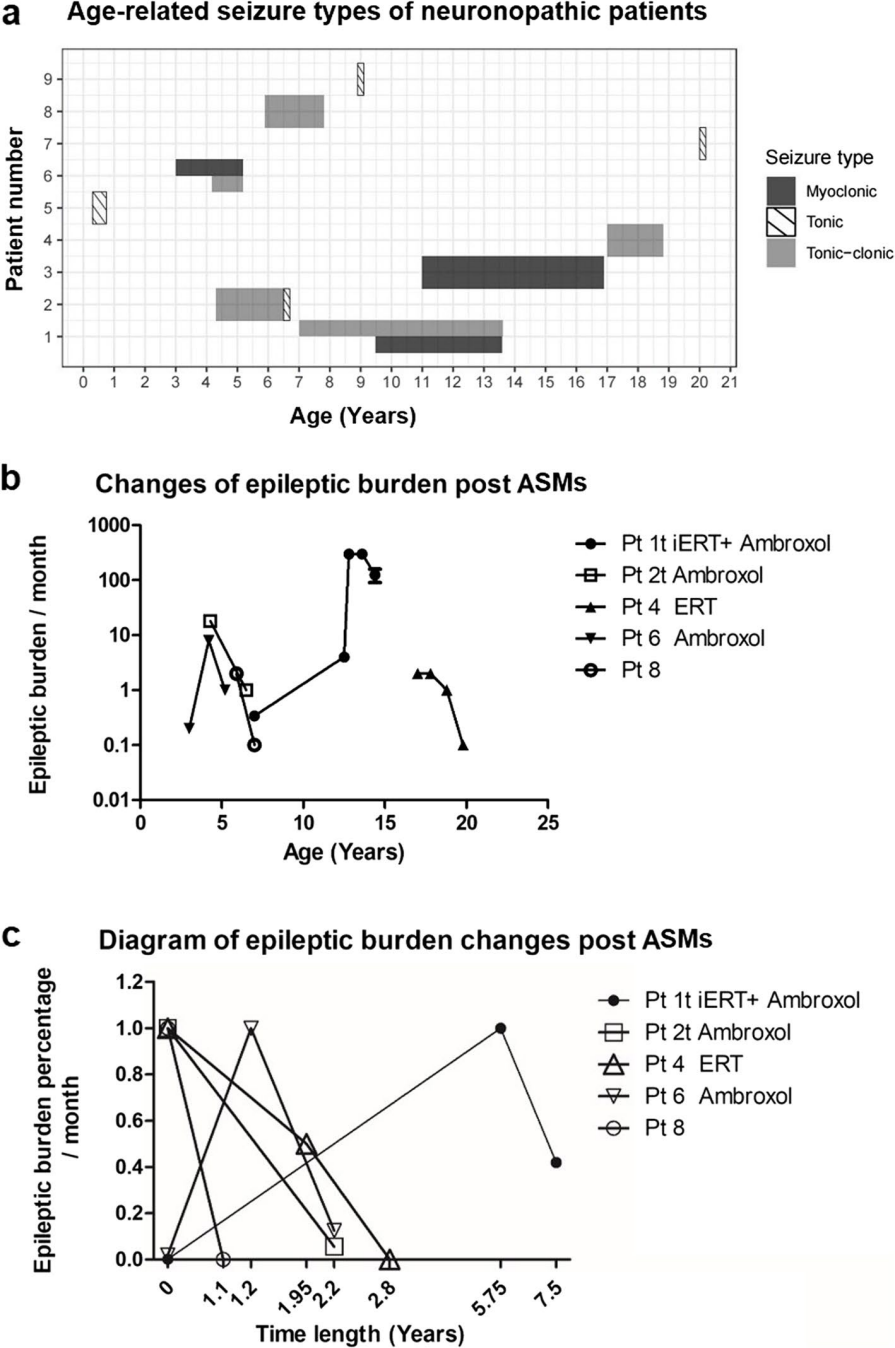
**a** Diagram of seizure type and age correlation for 9 neuronopathic patients with epilepsy; **b** Age-related changes of epileptic burden in GD patients treated with oral ASMs (In cases of patients with two mixed seizure types, the most significant changes in one type were assessed. For Patient 1 and 2, tonic-clonic seizures were chosen, marked as “t”, while for Patient 6, myoclonic seizures were selected to document the epileptic burden); **c** Graphical changes of epileptic burden in each GD patients with oral ASMs therapy (the most frequent seizure burden was 1 and other time points showed the percentage when compared with the highest burden)

Among the nine neuronopathic patients with epilepsy, the relation between type of seizures and age is shown in Fig. 4a. Three patients had a mixed seizure type. In terms of occurrence frequency, five patients (55.6%, 5/9) experienced tonic-clonic seizures, four had tonic seizures and three had myoclonic seizures. Two patients had epilepsy onset in adulthood, and they began regular enzyme replacement therapy (ERT) at the age of six (Patient 4 and 7).

Five patients received regular oral ASM therapy, with four of them also undergoing oral ambroxol treatment and regular or intermittent ERT. During follow-up, two patients achieved seizure-free for three years (Patients 2 and 8). Both of them initiated ASM treatment immediately after the first seizure attack, which was accompanied by EEG abnormalities. One of them had first seizure identified through EEG monitoring, while the other underwent EEG monitoring immediately after the occurrence of the initial seizure episode. Besides them, one patient remained seizure-free for one year, and the other two patients experienced remission (Patient 1 who was diagnosed as refractory epilepsy and Patient 6), with over 50% reduction of seizure frequency (Fig. 4b, c). No significant adverse effects of ASMs were observed in these patients.

Among the 9 neuronopathic patients with epilepsy, 7 patients completed more than twice of EEG monitoring, resulting in a total of 32 recordings. In six of them, EEG monitoring showed a trend of progressive aggravation, despite some clinical improvements. The EEG evolution included a significant increase of abnormal discharges, the development of SWC, polyspike-wave complex, or fast activities, and spread of discharges from a focal area to bilateral multiple foci. Furthermore, the appearance of discharges shifted from occurring solely during sleep to at both awake and sleep states. Among the most severe patients (Patients 1, 2, and 6), spikes or SWC rhythms were observed, with bursts or prolonged release. One patient exhibited ESES, and another showed disappearance of background rhythm and disturbance of sleep structure (Patient 6). Patient 4 showed clinical improvement, with a tendency of decrease of abnormal discharges in EEG.

(5) EEG patterns of ocular phenotypes

Among the 12 neuronopathic patients, one (Patient 12) presented solely with ocular symptoms, including oculomotor apraxia and strabismus. Three patients presented with both ocular symptoms and epilepsy. Another patient had walking instability.

**Fig. 5:**
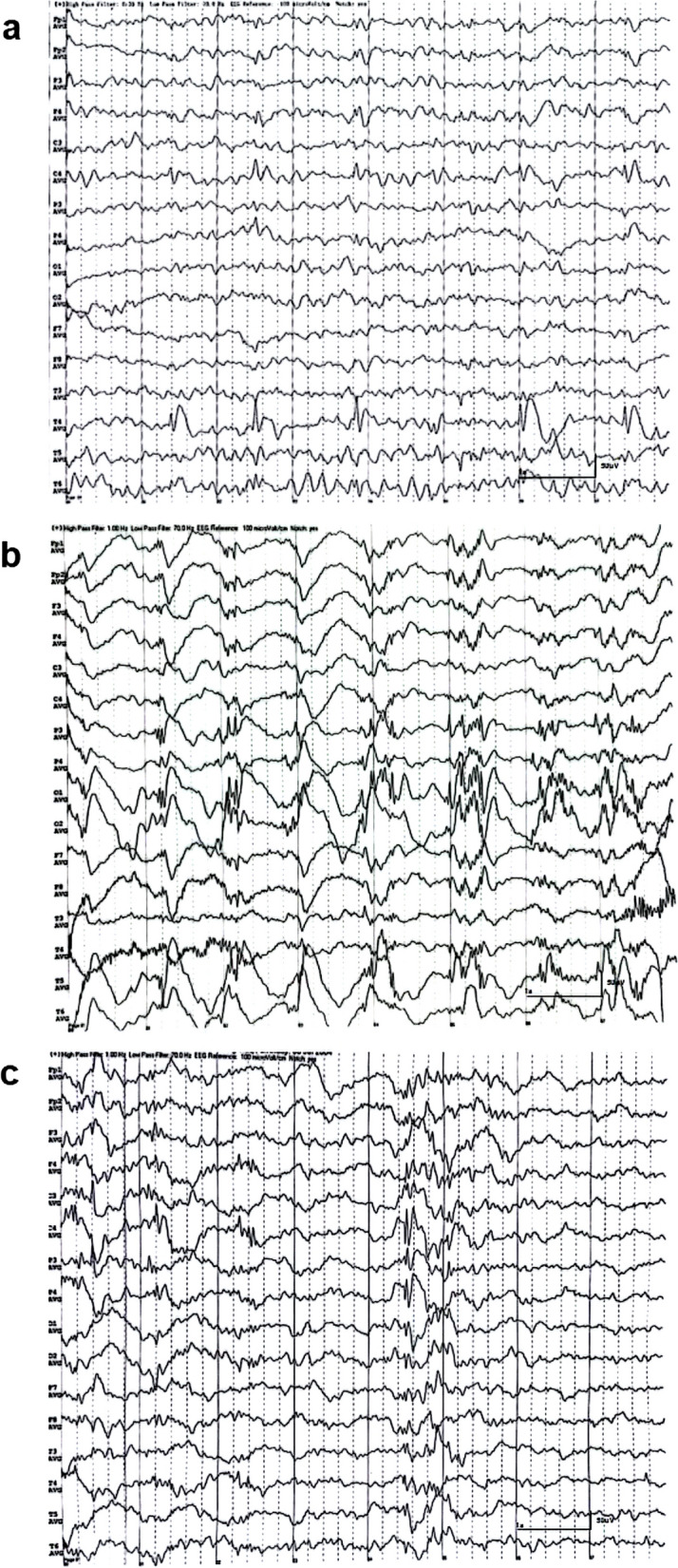
**a** EEG of one patient with ocular phenotype solely: during sleep there were sporadic or continuous low amplitude SWC discharges in region of right central, medial and posterior temporal lobe, medial temporal more pronounced. **b** EEG with oculomotor apraxia and newly onset epilepsy: sporadic or continuous low-amplitude SWC, polyspike and wave complex discharges were seen in the region of bilateral parietal, occipital, posterior temporal lobe. **c** EEG of transitional patient (Patient 12) who reported initial seizure: there was a burst of polyspike and wave complex, spikes in bilateral central, parietal regions, spreading to other leads. Note: an average referential montage was applied, the electrodes of EEG documents from top to bottom are marked as follows: Fp1 (left frontal pole), Fp2 (right frontal pole), F3 (left frontal), F4 (right frontal), C3 (left central), C4 (right central), P3 (left parietal), P4 (right parietal), O1 (left occipital), O2 (right occipital), F7 (left anterior temporal), F8 (right anterior temporal), T3 (left mid-temporal), T4 (right mid-temporal), T5 (left posterior temporal), T6 (right posterior temporal)

In the Patient 12 with ocular symptoms only, EEG showed sporadic or continuous unilateral SWC during sleep (Fig. 5a). Among the three epileptic patients with ocular symptoms, unilateral or bilateral SWC was observed during sleep.

The patient (Patient 11) with oculomotor apraxia and walking instability exhibited an epileptic pattern on EEG (Fig. 5b). The initial epileptic episode of this patient had been reported.

Two transitional patients (Patients 12 and 13) displayed EEG patterns characteristic of the epileptic phenotype. During both sleep and awake states, bursts or frequent paroxysmal SWC and polyspike-wave complexes were observed (Fig. 5c). Both patients reported occurrence of their initial seizures. One patient had a history of febrile seizures at the age of 13 and had a positive family history of GD (Patient 12).

Two neuronopathic patients had normal EEG recordings. One was a GD3 patient boy who initially presented with hepatosplenomegaly and oculomotor apraxia at 18 months. With intermittent ERT, his EEG monitoring remained normal at the age of 10. The other case was a female GD2 infant who developed splenomegaly at two months of age. By 10 months, she exhibited developmental delay and oculomotor apraxia. Her EEG monitoring at age 1 year was normal.

## Discussion

In this study, half of the GD patients were found to have EEG abnormalities. The common EEG feature of these patients was generalized or bilateral multi-focal low-amplitude SWC or sharp waves, which is consistent with previous empirical findings [9, 11]. We observed distinct interictal EEG patterns that corresponded to different clinical phenotypes. In neuronopathic GD patients with epilepsy, EEG abnormalities included SWC during both awake and sleep states mainly in the bilateral Rolandic areas and less frequently in other lobes. About one-third of the GD1 patients were of the transitional type, and their risk of developing into the GD3 type increased with enhanced EEG abnormality. The EEG of the transitional GD patients was characterized by unilateral or bilateral sharp waves with predominant involvement of the frontal lobe. Only the occipital lobe was not involved at all.

### EEG patterns and EEG severity progression

Our study revealed that the transitional patients who exhibited EEG patterns resembling those of neuronopathic patients with epilepsy, such as generalized bilateral polyspikes or polyspike-wave complex during both awake and sleep states, had an increased risk of experiencing clinical episodes. This finding supports the potential use of EEG as a tool to predict the risk of clinical seizure episodes in GD1 patients. In fact, in our study, EEG successfully predicted the onset of epilepsy in three patients several years before their initial clinical episodes occurred. This highlights the clinical utility of EEG in identifying high-risk patients to facilitate early intervention. In particular, we found that EEG monitoring can play a crucial role in the early detection and intervention of neurological manifestations in transitional GD1 patients without any clinical symptoms of central nervous system (CNS) involvement. This "sentinel effect" allows clinicians to closely monitor EEG patterns and make timely adjustments to the treatment plan for GD patients.

Based on our experience, we recommend EEG monitoring at intervals of 6–12 months for transitional GD1 patients who do not show any clinical signs of CNS involvement. This regular EEG monitoring provides an opportunity to detect any emerging abnormalities and enables prompt intervention to optimize treatment outcomes for these patients.

**Fig. 6:**
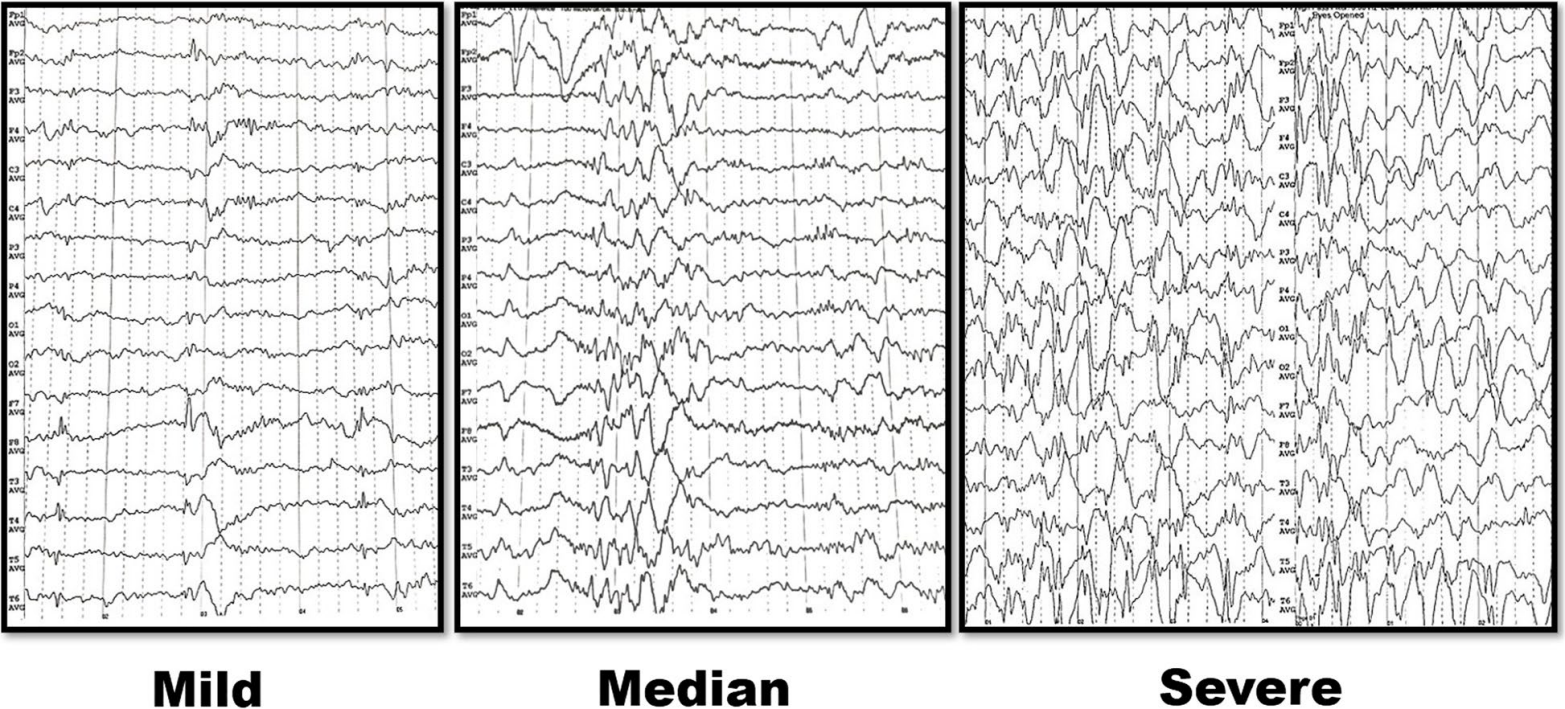
A dynamic evolution from mild to severe in the EEG manifestation of GD patients. Note: an average referential montage was applied, the electrodes of EEG documents from top to bottom are marked as follows: Fp1 (left frontal pole), Fp2 (right frontal pole), F3 (left frontal), F4 (right frontal), C3 (left central), C4 (right central), P3 (left parietal), P4 (right parietal), O1 (left occipital), O2 (right occipital), F7 (left anterior temporal), F8 (right anterior temporal), T3 (left mid-temporal), T4 (right mid-temporal), T5 (left posterior temporal), T6 (right posterior temporal)

It has been suggested that GD1 transforming into GD3 is a dynamic process [5, 12]. Our study supported this theory again. Similar to the clinical course, the EEG manifestation of GD patients also presents a dynamic evolution from mild to severe: unilateral or bilateral discharges at sleep in the mild stage, unilateral to bilateral various abnormal discharges during both awake and sleep states in the moderate stage; and eventually disappearance of background rhythm and disruption of the sleep cycle in the severe stage (Fig. 6). According to the current data, the evolution of waveform from sharp waves to SWC, polyspike-wave complex, spike rhythm or fast activity, and generalization from unilateral to bilateral multi-focus are EEG signs for a poor prognosis.

### EEG patterns and ASMs

In this study, the neuronopathic GD patients with epilepsy had a good response to oral ASMs when administered promptly and appropriately, leading to a reduced seizure burden or a seizure-free status lasting for years. This outcome is very encouraging as it challenges the conventional understanding of the neuronopathic GD with epilepsy [4, 5, 8]. A key factor contributing to the optimistic outcomes is the timely, appropriate intervention provided by regular EEG monitoring, which is more sensitive than clinical observations alone. This highlights the advantages of EEG in evaluating neurological damage in GD patients, as observed in previous studies [10, 13]. The significance of this study lies in the finding that EEG is capable of monitoring disease progression and serves as an early warning system for seizure development. This offers an opportunity for early intervention, resulting in effective control of epilepsy. Moreover, EEG monitoring is convenient, particularly in light of the pandemic situation. Clinicians can closely monitor patients’ condition as long as standard EEG equipment is available, regardless of their geographical location.

Based on these findings, it is recommended that neuronopathic GD patients undergo EEG monitoring every 3–6 months, with more frequent monitoring in severe cases. So far, the indication for ASMs is the presence of at least one clinical seizure accompanied by an epileptic EEG pattern. However, the optimal timing for starting ASMs in these patients is still a topic under discussion. Future research will further investigate the potential benefits of initiating anti-epileptic therapy in GD patients with moderate or severe EEG abnormalities without clinical episodes. Additionally, our study observed that some GD3 patients who did not receive ASMs did not experience a significant increase of seizure burden, and some even showed a tendency of decrease, although their EEG abnormalities were still aggregating. This could be attributed to the genetic heterogeneity of GD3, which is an interesting direction worth investigating further.

Briefly, the identification of specific subgroups of GD patients who require prompt ASMs and who are eligible for a suspension of ASMs will be the focus of our upcoming research. Understanding these distinctions, particularly within a genetic dimension, will guide treatment for each subgroup, ultimately improving the management of GD-related epilepsy. Once the GD patients start taking ASMs, they must maintain a life-long treatment as the CNS injury is irreversible, and their EEG patterns will not return to normal thereafter.

### EEG patterns and ERT

Traditionally, ERT has been considered to be less efficacious in controlling or improving CNS pathology in GD due to the blood-brain barrier [8, 14, 15]. However, our study suggests that application of ERT or ambroxol may delay the onset of epilepsy in GD patients. Some recent pioneer studies also indicated a positive effect of ERT [16, 17]. Patient 4, the GD3 patient, exhibited easy-to-control seizures together with improvement in EEG recordings following ERT. This patient also had mild neurological symptoms that developed from adulthood. These observations suggest that ERT may play a beneficial role in resisting or reducing neurological damage by improving the microenvironment of brain cells. Furthermore, another GD3 patient receiving ERT in our study had only oculomotor nerve damage, with normal EEG findings throughout the follow-up. This finding further supports the microenvironment-associated neuroprotective effect of ERT in preventing or minimizing neurological damage in GD patients. Further research is required to fully understand the underlying mechanisms and the extent of these benefits.

Unfortunately, although seizures were effectively controlled by early application of ASMs in combination with primary interventions such as ERT, the overall course of disease progression and the deterioration of EEG were not reversed in some neuronopathic patients. These patients and their families must cope with other challenges such as developmental delay, ataxia, parkinsonian tremor [15, 18]. The situation may become even more daunting if their epilepsy remains uncontrolled. This highlights the intractable nature of neuronopathic GD and our journey to address it continues.

### EEG patterns and pathophysiology

Our analysis revealed that abnormal EEG manifestations often appeared earlier and recovered later than the clinical symptoms. This discrepancy may be attributed to the difficulty in clearing excessive glucosylceramide and glucosylsphingosine caused by glucocerebrosidase deficiency in the CNS [19, 20]. We also found that the abnormal discharges in EEG began in the anterior and frontal lobes, progressed to the bilateral Rolandic areas, and ultimately affected the occipital lobe. This pattern contradicts the typical developmental trajectory of white matter in children. These findings suggest that the less mature the brain is, the more susceptible it is to damage by the accumulation of abnormal glucosylceramide and glucosylsphingosine. These observations provide valuable insights into the pathophysiology of GD-related neurologic damage and further emphasize the importance of early intervention to mitigate disease progression.

In this study, we observed that patients with strabismus and oculomotor apraxia exhibited unilateral or bilateral abnormal discharges during sleep in their EEG recordings. This finding is distinct from the typical epileptic phenotype associated with GD. We hypothesize that the underlying pathological mechanism of oculomotor abnormalities is different from that of brain involvement. In alignment with insights from the literature, gliosis and neuronal loss observed in the basal ganglia and brainstem, rather than selective neuron and glial cell death in the cerebellum, may contribute to the oculomotor damage [20–22]. Further research is needed to better understand the distinct pathological processes that contribute to different manifestations of the disease.

### EEG patterns and other important questions

We have observed an interesting correlation between EEG findings and GD2 phenotypes, again highlighting the genotypic and phenotypic heterogeneity within GD [23]. A GD2 patient exhibited normal EEG, despite having severe clinical manifestations. This finding is consistent with a previous case report of another GD2 patient with normal EEG despite severe neurological symptoms [10]. It is notable that both patients had their EEG monitored at 1 year of age.

In contrast, we observed a very severe GD2 patient who exhibited abnormal EEG patterns starting at eight months of age (Patient 5). Furthermore, we noticed an infant case of GD1 (not included in this study) who had a similar age of onset, presenting a very mild EEG abnormality at 15 months old.

Based on these observations, we speculate that the general age for EEG detection of nervous system damage caused by accumulated glucosylceramide and glucosylsphingosine is around 1 year of age, except in some extremely severe cases. EEG monitoring in infants may provide more valuable insights into the neurological impact of GD regarding different clinical subtypes.

It is observed that GD1 patients with normal EEG recordings had a later onset age compared to transitional patients, whose onset age ranged between one and two years. We did not observe any significant differences between these two groups in other general aspects.

Based on the above findings, we speculate that the younger the child, the less mature the blood-brain barrier; therefore, excessive accumulation of glucosylceramide and glucosylsphingosine results in more severe damage to the immature nervous system. This suggests that EEG monitoring should be more actively applied to younger patients to detect and assess neurological involvement. However, this needs to be verified by more data from younger patients. More evidence is needed to fully understand the relationship between the age of onset, the blood-brain barrier maturity, and the severity of nervous system damage in GD.

We observed no correlation between splenectomy and EEG manifestations in this study. This suggests that splenectomy does not seem to have a direct impact on EEG findings in GD, at least in the context of our study.

### Limitations

However, a limitation of this study is the single-center design, which may introduce institution-specific biases. Therefore, studies with larger sample sizes comprising a greater representation of transitional and younger neuronopathic patients, are essential to validate and enhance the robustness of the findings.

## Conclusions

In summary, regular standard EEG monitoring can help identify GD1 patients at high risk of developing into GD3, allowing for earlier intervention with ASMs and primary therapies to GD. This facilitates management of seizures and improves the prognosis, with younger children likely to benefit more. Primary therapies such as ERT and ambroxol, as well as other emerging drugs, combined with EEG monitoring and ASMs, can enhance the outcomes for neuronopathic GD patients with epilepsy.

Systematic clinical studies on EEG and prognosis in GD patients pose significant challenges due to the rarity and sporadic nature of GD, as well as a high rate of misdiagnosis and mortality associated with the disease. Nevertheless, collaborative efforts have opened up new directions, and it is hoped that more multidisciplinary experts join these efforts to bring additional benefits to GD patients.

## Data Availability

The datasets used and/or analysed during the current study are available from the corresponding author on reasonable request.

## Abbreviations

ASMs: Anti-seizure medications
EEG: Electroencephalogram
AEEG: Ambulatory EEG
ERT: Enzyme replacement therapy
ESES: Electrical Status Epilepticus during Sleep
GD: Gaucher disease
GD1: Type 1 Gaucher disease
GD2: Type 2 Gaucher disease
GD3: Type 3 Gaucher disease
ILAE: The International League Against Epilepsy
PME: Progressive myoclonic epilepsy
SWC: Spike-and-wave complex
VEEG: Video EEG
